# Impact of prenatal environmental exposure on offspring neurodevelopment and susceptibility to neurodegenerative diseases: mechanisms and perspectives

**DOI:** 10.3389/fpubh.2026.1837731

**Published:** 2026-07-07

**Authors:** Jiajie Yu, Hongmei Lian, Mengjiao Yang, Shenglin Hu, Yunxia Zhou, Jin Cheng, Yali Wang

**Affiliations:** 1 Department of Nursing, Ya'an People's Hospital, Yaan, China; 2 Department of Cardiac and Great Vascular Surgery, Affiliated Hospital of North Sichuan Medical College, Nanchong, China

**Keywords:** developmental origin of health and disease, disease susceptibility, epigenetic modification, neurodegenerative diseases, neurodevelopment, neuroinflammation, prenatal environmental exposure

## Abstract

Pregnancy constitutes a critical window for fetal nervous system development. Maternal environmental exposure can trigger placenta-mediated intrauterine perturbations, exerting persistent programming effects on fetal neural development and elevating the susceptibility to neurodegenerative diseases in adulthood. This review systematically summarizes typical prenatal exposure types, including air pollutants, heavy metals, endocrine-disrupting chemicals, nutritional imbalance, and maternal stress. Focusing on four core mechanistic pathways—epigenetic modification, oxidative stress, neuroinflammation, and mitochondrial dysfunction—this study integrates epidemiological evidence, animal model data, and molecular mechanistic findings to elaborate how early-life environmental exposure reshapes neurodevelopmental trajectories and mediates long-term neurological damage. Notably, this review highlights the interactive feedback and cascade amplification effects among multiple biological mechanisms, and strictly distinguishes well-established causal associations from speculative inferences. This work constructs a stratified evidence framework for the developmental origin theory of neurodegenerative diseases, providing scientific support for precise pre-pregnancy and prenatal disease prevention and intervention strategies.

## 1 Introduction

Prenatal environmental exposure refers to a variety of exogenous factors that mothers encounter during gestation, which can directly disrupt the fetal internal environment across the placental barrier (1). The fetal nervous system undergoes rapid proliferation, migration, differentiation, and synaptogenesis throughout pregnancy, rendering it extremely vulnerable to environmental perturbations (2). Accumulating evidence demonstrates that early-life environmental exposure not only induces adverse neurodevelopmental outcomes in childhood (e.g., cognitive and behavioral abnormalities) but also increases the risk of neurodegenerative diseases in adulthood via developmental programming, which further extends the developmental origins of health and disease (DOHaD) hypothesis in neuroscience (3). Clarifying how prenatal environmental exposure sculpts long-term neurological health trajectories is essential for the early-life prevention of neurodegenerative disorders (4).

The fetal stage is a decisive period for central nervous system maturation, during which environmental disturbances permanently shape the structure and function of the developing brain (5). A wide spectrum of environmental stressors, including chemical toxicants, pharmaceutical agents, pathogen infection, nutritional imbalance, and psychological stress, can profoundly alter fetal neurodevelopment. For instance, prenatal alcohol exposure is the primary cause of fetal alcohol spectrum disorders, characterized by persistent cognitive, behavioral, and physical impairments (6). Alcohol toxicity disrupts mitochondrial function, exacerbates oxidative stress, and impairs cellular respiration, thereby inducing irreversible structural and functional abnormalities in the developing brain (6). Similarly, excessive prenatal glucocorticoid exposure, resulting from either maternal psychological stress or exogenous glucocorticoid administration, exerts long-term detrimental effects on fetal brain development and correlates with elevated risks of neurological disorders (5). Maternal immune activation triggered by gestational infection or inflammatory responses serves as a pivotal risk factor for offspring behavioral deficits and neuropsychiatric diseases, which substantially disrupts fetal brain development by enhancing neural progenitor cell proliferation and inhibiting core neurodevelopmental signaling pathways (7).

In addition to direct neurotoxic environmental substances, gestational pharmaceutical exposure has attracted widespread research attention. Prenatal treatment with selective serotonin and norepinephrine reuptake inhibitors disrupts maternal and placental serotonin signaling, impairs fetal neurodevelopment, and increases the susceptibility to gastrointestinal disorders in children, implying potential crosstalk along the gut–brain axis (8). Prenatal opioid exposure induces genome-wide DNA methylation alterations in the placenta, which disturbs epigenetic regulation of genes associated with synaptic structure and neural development, ultimately leading to fetal brain dysplasia and neonatal abstinence syndrome (9). Furthermore, prenatal nicotine exposure remodels epigenetic profiles of germ cells and alters DNA methylation levels of functional genes involved in peripheral nervous system signaling cascades (10).

Environmental pollutants represent another major threat to fetal neurodevelopment. Prenatal exposure to air pollutants, particularly fine particulate matter, is closely associated with fetal growth restriction and impaired neurodevelopment, primarily attributable to placental barrier dysfunction induced by elevated cortisol levels (11). Heavy metal pollutants, including lead, manganese, mercury, arsenic, and cadmium, cause persistent adverse impacts on childhood neurodevelopment, especially in low- and middle-income regions (12). Emerging per- and polyfluoroalkyl substances are linked to declined cognitive, motor, and linguistic abilities as well as increased behavioral problems in children (13). Nanoparticles can penetrate the placental barrier and trigger fetal developmental retardation and malformations via oxidative stress, neuroinflammation, and dysregulated gene expression (14). Prenatal pyrethroid pesticide exposure exhibits specific developmental neurotoxicity, which impairs fetal neurodevelopment principally through disrupting thyroid hormone homeostasis (15).

Furthermore, maternal psychosocial factors are indispensable confounding and regulatory variables in fetal neurodevelopment. Prenatal maternal psychological distress alters offspring neurodevelopmental trajectories, affects autonomic nervous system function reflected by fetal heart rate indicators, and correlates with infant temperament characteristics (16). Gestational stress exposure impairs the stress regulatory capacity and resilience of the offspring autonomic nervous system (17). Notably, positive parental sensitivity can buffer the adverse associations between prenatal stress exposure and postnatal physiological responses and temperament, thereby facilitating the development of infant stress resilience (18).

The neurodevelopmental impairments induced by early prenatal environmental exposure can persist into adulthood and heighten the susceptibility to neurodegenerative diseases. Developmental exposure to the environmental neurotoxin β-methylamino-L-alanine triggers long-term cognitive dysfunction and neurodegeneration in rodent models (19). Prenatal alcohol exposure exacerbates the vulnerability to Alzheimer's disease-related pathological changes in later life by inducing sustained neuroinflammation and remodeling the endogenous cannabinoid system (20). Prenatal diesel exhaust particle exposure impairs hippocampal synaptic plasticity, thus compromising learning and memory functions in offspring (21). These findings strongly support the DOHaD hypothesis, confirming that prenatal environmental exposure lays the pathological foundation for late-life neurodegenerative diseases via epigenetic programming and other long-term regulatory mechanisms (22). Therefore, it is critical to comprehensively explore the classification of prenatal environmental stressors, their immediate and long-term neurological impacts, underlying biological mechanisms, and translational research progress.

## 2 Classification and characteristics of major prenatal environmental exposures

### 2.1 Chemical pollutant exposure

Prenatal chemical pollutant exposure is a critical environmental factor affecting offspring neurodevelopment and long-term susceptibility to neurodegenerative diseases. These ubiquitous pollutants enter the maternal circulation via diverse routes and perturb core biological processes governing fetal development. As the most common gestational chemical stressors, air pollutants, especially fine particulate matter (PM2.5), are closely linked to multiple adverse pregnancy outcomes, including placental dysfunction and preeclampsia (23). A population-based study of Chinese pregnant women verified that first-trimester PM2.5 exposure was significantly correlated with decreased maternal free triiodothyronine (FT3) and free thyroxine (FT4) levels, suggesting that air pollution disrupts maternal endocrine homeostasis, thereby interfering with fetal developmental programming (24).

Heavy metals constitute another category of well-validated neurotoxic pollutants that readily cross the placental barrier and directly target the developing fetal nervous system. Systematic reviews have confirmed that prenatal heavy metal exposure increases the risk of hypertensive disorders of pregnancy (HDP), with lead exposure presenting the most robust causal evidence (25). Placental accumulation of heavy metals induces fetal growth restriction, and their neurotoxic effects exhibit distinct dose-response relationships and gestational window specificity (26).

Endocrine-disrupting chemicals (EDCs) are exogenous compounds that mimic or antagonize endogenous hormonal signaling, posing unique and severe threats to fetal brain development. Bisphenol A (BPA) and phthalates (PAEs) are the most prevalent EDCs detected in human biomonitoring studies. Prenatal phthalate exposure disrupts maternal thyroid hormone secretion in a non-linear inverted U-shaped dose–response pattern (27). Given the indispensable role of thyroid hormones in fetal cortical morphogenesis and neural maturation, thyroid hormonal disturbance inevitably triggers cortical structural abnormalities and persistent cognitive deficits throughout life. Furthermore, benzophenones and other emerging EDCs are ubiquitously detectable in pregnant populations, with exposure levels tightly associated with personal care product application and dietary habits, further disrupting fetal developmental homeostasis (28). Notably, these chemical pollutants seldom occur as single exposures; combined co-exposures commonly exert synergistic toxic effects, substantially amplifying the risk of adverse neurodevelopmental outcomes (Table 1).

**Table 1:** Types and neurotoxic effects of major chemical pollutants exposure during pregnancy.

Pollutant category	Typical representatives	Exposure routes	Core neurotoxic manifestations	Toxicological characteristics
Air pollutants	Fine particulate matter (PM2.5), polycyclic aromatic hydrocarbons	Respiratory inhalation	Placental barrier injury, thyroid hormone disruption, and abnormal brain regional volume	The fetal brain is susceptible throughout gestation, with markedly higher vulnerability in the first trimester
Heavy metals	Lead, mercury, arsenic, cadmium	Dietary intake and environmental contact	Fetal growth restriction and aberrant proliferation of neural progenitor cells	Exhibits a prominent and consistent dose–response relationship
Endocrine-disrupting chemicals	Bisphenol A, phthalates	Dietary intake, personal care products, and plastic packaging materials	Thyroid hormone disturbance and dysregulated epigenetic modification	Presents a typical non-linear dose–response pattern
Emerging pollutants	Per- and polyfluoroalkyl substances (PFAS)	Dietary intake and drinking water ingestion	Impaired cognitive and motor development and increased risk of neurobehavioral disorders	Possesses strong biological accumulation and persistent toxicity
Pesticides	Pyrethroids	Dietary intake and environmental exposure	Impaired thyroid hormone function and damaged synaptic plasticity	Exerts specific and targeted developmental neurotoxicity

### 2.2 Physical and psychosocial environmental exposures

In addition to chemical toxicants, prenatal exposure to physical and psychosocial stressors also exerts profound and long-lasting impacts on fetal neurodevelopment. Maternal nutritional status represents a critical physical environmental determinant. Both malnutrition and overnutrition during gestation can reshape fetal metabolic homeostasis and disrupt developmental programming. Notably, although healthy dietary patterns (e.g., Mediterranean diet) benefit maternal cardiovascular and metabolic health, certain nutrient-dense foods serve as major dietary sources of persistent environmental chemicals, including polychlorinated biphenyls (PCBs) and per- and polyfluoroalkyl substances (PFAS). Consequently, pregnant women adhering to healthy dietary regimens may exhibit elevated circulating levels of these toxic pollutants (29). This paradoxical phenomenon highlights an unrecognized public health dilemma: the promotion of healthy prenatal diets should be complemented by targeted strategies to reduce chemical contamination within the food chain.

Maternal psychological stress and emotional status constitute pivotal psychosocial exposure factors. Chronic gestational stress elevates maternal glucocorticoid levels, which impair placental barrier function, reprogram fetal hypothalamic–pituitary–adrenal (HPA) axis development, and permanently alter lifelong stress reactivity. Such intrauterine programming is closely linked to increased susceptibility to emotional disorders and reduced hippocampal volume in offspring (30). Furthermore, prenatal infection and inflammatory conditions serve as crucial mediators bridging physical exposure and immune dysregulation. Maternal pathogen infection (e.g., influenza virus) or sterile inflammatory activation triggers robust maternal immune responses, induces placental and fetal pro-inflammatory cytokine release, and initiates persistent fetal neuroinflammation. This inflammatory intrauterine microenvironment impedes oligodendrocyte maturation and myelination, a fundamental process ensuring rapid and efficient neural signal transmission.

Although current clinical guidelines regarding prenatal environmental safety are largely opinion-based rather than evidence-driven, accumulating evidence confirms that exposure to natural environments optimizes pregnancy outcomes and maternal mental well-being, while extreme environmental conditions impose additional gestational risks (31). Traditional exposure assessment approaches relying solely on residential address fail to capture dynamic daily activity patterns, leading to potential measurement bias. Advanced dynamic monitoring tools, such as global positioning system (GPS)-based spatial tracking, are therefore recommended for accurate exposure quantification in prenatal cohort studies (32).

## 3 Immediate and long-term neurodevelopmental consequences of prenatal environmental exposures

### 3.1 Alterations in brain structural and functional development

Prenatal environmental exposures induce multidimensional alterations in offspring brain development, spanning macroscopic anatomical remodeling to microscopic neural circuit dysfunction. Neuroimaging studies have provided robust human evidence linking gestational toxicant exposure to region-specific brain morphological abnormalities. Prenatal and early postnatal exposure to ambient air pollutants, including nitrogen oxides, polycyclic aromatic hydrocarbons (PAHs), and fine particulate components, significantly modulates regional brain volume (33). Higher pollutant concentrations are correlated with reduced corpus callosum and hippocampal volume, as well as enlarged amygdala, nucleus accumbens, and cerebellar volume in children (33). Another prospective study demonstrated that prenatal coarse particulate matter (PMcoarse) exposure increases cortical gray matter volume in childhood, with this association being more pronounced in children with high polygenic risk scores for Alzheimer's disease, indicating that genetic background modulates air pollution-induced structural brain alterations (34). These deviated morphological phenotypes reflect either delayed neurodevelopmental maturation or persistent stress-induced developmental disruption (33).

Additionally, prenatal tobacco smoke exposure is associated with reduced total brain volume, diminished gray and white matter volume, and decreased cortical surface area and gyrification in prepubertal offspring. These adverse structural alterations are predominantly observed in children with sustained gestational smoke exposure rather than first-trimester-only exposure (35). Collectively, neuroimaging evidence validates that prenatal adverse exposures disrupt the structural integrity of core brain regions governing higher-order cognition and emotional regulation.

Epidemiological cohort studies have extensively validated the associations between diverse prenatal environmental exposures and adverse neurobehavioral outcomes in offspring. A prospective mother–infant cohort study based in Tianjin, China (172 pairs) demonstrated that first-trimester PAH mixture exposure was negatively correlated with personal-social ability and language development scores in infants aged 6–12 months (36). Although gestational exposure to glyphosate and its metabolite aminomethylphosphonic acid (AMPA) showed no significant associations with cognitive, social, or behavioral performance in children aged 3–4 years, a marginal negative trend was observed between AMPA concentrations and performance IQ (37). The neurotoxic effects of prenatal lead exposure are more definitive: prenatal lead burden is associated with delayed neurodevelopment at 18 months of age, characterized by lower Ages & Stages Questionnaire (ASQ) total scores, as well as impaired fine motor and problem-solving abilities, particularly in male infants (38). Data from the Shanghai Birth Cohort further indicated that co-exposure to lead and prenatal stress exerts synergistic detrimental effects on infant neurodevelopment, especially socioemotional competence, which are more severe than single exposure (39). Furthermore, prenatal PFAS exposure is strongly implicated in multiple adverse pediatric neurodevelopmental phenotypes, including psychomotor retardation, externalizing behavioral problems, and comprehensive developmental deficits (40). These findings collectively confirm that prenatal environmental stressors increase the lifelong risks of attention-deficit/hyperactivity disorder (ADHD), autism spectrum disorder (ASD), cognitive impairment, and emotional dysregulation in offspring.

Animal models have further elucidated the cellular and molecular mechanisms by which prenatal toxicant exposure impairs synaptic plasticity and neural circuit maturation. For instance, gestational and lactational exposure to deltamethrin, a typical pyrethroid pesticide, induces persistent learning, memory, and motor dysfunction in male offspring (41). Mechanistically, deltamethrin exposure downregulates hippocampal M1 muscarinic acetylcholine receptor expression, suppresses the downstream AKT/mTOR signaling cascade, inhibits dynamic GluA1 trafficking, and reduces hippocampal dendritic spine density. Meanwhile, deltamethrin toxicity blunts BDNF/TrkB signaling and impairs dendritic arborization of cerebellar Purkinje cells (41).

In addition, prenatal polystyrene nanoplastics exposure markedly reduces fetal cortical thickness and disrupts neocortical neuronal migration, characterized by excessive proliferation of superficial-layer neurons and diminished deep-layer neuronal populations. Ultrastructural analysis reveals widened synaptic clefts and decreased postsynaptic density in the hippocampus (42). These microstructural abnormalities contribute to increased anxiolytic-like behaviors and impaired spatial memory in adolescent offspring (42). Prenatal diesel exhaust particle (DEP) exposure also alters the expression of synaptic plasticity- and learning memory-related proteins (including CPEB3, NMDAR subunits, PKA, SYP, PSD95, and p-CREB) in the male offspring hippocampus, thereby permanently compromising synaptic function (21). These preclinical findings verify that prenatal environmental insults persistently alter hippocampal long-term potentiation, prefrontal cortical synaptic density, and neurotransmitter homeostasis, ultimately disrupting cognitive processing and reward circuit function across the lifespan.

### 3.2 Developmental programming of neurodegenerative disease susceptibility

Adverse prenatal environmental exposures initiate premature aging phenotypes and lay the pathological foundation for late-life neurodegeneration via multi-layered programming mechanisms. Telomere attrition, a hallmark of cellular senescence, is profoundly modulated by intrauterine environmental conditions. Prenatal high-carbohydrate and high-fat diets, gestational alcohol exposure, triclosan contact, and maternal sleep-disordered breathing are significantly associated with shortened offspring telomere length (43). While the evidence regarding maternal stress and smoking remains inconsistent, adequate gestational nutrition (e.g., vitamin C, vitamin D, folate, and magnesium supplementation) and Mediterranean dietary patterns contribute to preserved placental telomere length (43).

Moreover, prenatal toxicant exposure induces cumulative oxidative damage and impairs endogenous DNA repair capacity. Gestational methylmercury exposure triggers robust oxidative stress in the rat offspring hippocampus and cerebellum, manifested by suppressed antioxidant capacity, reduced glutathione levels, and elevated lipid peroxidation and nitrosative stress (44). *In vitro* models further demonstrate that oxidative stress substantially remodels the transcriptomic profiles of signaling pathways associated with neurodevelopmental disorders (e.g., PI3K/Akt and axon guidance pathways), suggesting that early-life redox imbalance disrupts normal neuronal differentiation and brain maturation, thereby elevating the vulnerability to neuropsychiatric disorders in adulthood (45). Persistent mitochondrial dysfunction, unresolved neuroinflammation, and chronic oxidative stress induced by prenatal adverse exposures are recognized as the core mechanistic links connecting developmental neurodevelopmental disruption with late-life neurodegenerative pathologies, including Alzheimer's disease (AD), Parkinson's disease (PD), and amyotrophic lateral sclerosis (ALS) (4). Accordingly, intrauterine environmental stressors accelerate brain aging and enhance susceptibility to age-related neurodegeneration.

Preclinical studies have validated that prenatal adverse exposures increase the propensity for pathological protein aggregation in the developing brain, recapitulating the early pathological features of neurodegenerative diseases. Aberrant β-amyloid (Aβ) accumulation and tau hyperphosphorylation represent the defining pathological hallmarks of AD. Although longitudinal human evidence directly linking prenatal exposure to these proteinopathies remains limited, mechanistic and animal studies provide compelling mechanistic insights. Prenatal DEP exposure not only impairs hippocampal synaptic plasticity but also disrupts neuronal protein homeostasis via modulating CPEB3-associated protein regulatory networks (21). Protein misfolding and aggregation are observed not only in neurodegenerative disorders but also in gestational complications such as preeclampsia, implying that the prenatal period is inherently vulnerable to proteostatic dysregulation (46).

Clinical evidence further supports the developmental programming of brain aging. Maternal depressive symptoms during pregnancy correlate with increased brain age discrepancy in young adult offspring, where structural brain age exceeds chronological age. Deviated neurodevelopmental trajectories (either accelerated or delayed maturation) predict increased risks of anxiety and emotional dysregulation (47). Accelerated brain aging is well-established as a reliable biomarker of neurodegenerative vulnerability. Notably, maternal gestational depression interacts with air pollution exposure trajectories to synergistically predict brain age deviation and long-term mental health outcomes in young adulthood (48). These findings indicate that prenatal environmental adversities reshape developmental trajectories and disrupt proteostatic balance, facilitating the progressive accumulation of neurodegenerative pathologies in later life.

Furthermore, prenatal neurotoxic or inflammatory insults specifically enhance the vulnerability of nigrostriatal dopaminergic neurons, thereby programming the susceptibility to PD, which is pathologically characterized by progressive loss of dopaminergic neurons in the substantia nigra pars compacta. Prenatal methylmercury exposure induces autism-like behavioral deficits in adult mice, driven by premature neuronal differentiation of cortical radial glial progenitors, indicating that early developmental perturbation permanently alters the survival fitness of specific neuronal subpopulations, including dopaminergic neurons (49). Prenatal corticosterone exposure significantly remodels brain transcriptomic signatures in offspring, with robust upregulation of neurodegeneration-related genes (50). Proteomic profiling of cerebrospinal fluid extracellular vesicles in adult rhesus monkeys with prenatal cocaine exposure reveals differential expression of proteins closely implicated in neuroinflammation and neurodegenerative pathogenesis (51).

These observations strongly support the “two-hit” hypothesis of neurodegenerative programming: prenatal environmental adversity serves as the first hit, rendering dopaminergic neurons developmentally vulnerable. Subsequent aging-related degeneration, environmental toxicant re-exposure, or chronic neuroinflammation in adulthood acts as the second hit, ultimately triggering irreversible neuronal loss and disease onset. Abnormal synaptic plasticity and persistent neuroinflammation observed in fetal alcohol spectrum disorders further validate that prenatal developmental perturbation establishes long-term neural circuit fragility (52). Collectively, prenatal adverse exposures reshape neurodevelopmental trajectories, induce chronic low-grade neuroinflammation, and initiate epigenetic reprogramming, rendering nigral dopaminergic neurons highly susceptible to age-related neurodegeneration.

## 4 Core mechanisms underlying prenatal exposure-mediated neurodevelopmental dysregulation and disease susceptibility

### 4.1 Epigenetic programming mechanisms

Prenatal environmental exposures exert persistent programming effects on offspring neurodevelopment and lifelong neurodegenerative susceptibility via diverse epigenetic modifications, which persist throughout postnatal maturation and adulthood. DNA methylation serves as a central regulatory mechanism. Gestational exposure to endocrine-disrupting chemicals (e.g., bisphenols and phthalates) perturbs the methylation landscape of key neurodevelopmental genes in the fetal brain (27). Prenatal bisphenol A exposure remodels the hippocampal transcriptome associated with AD and ASD pathogenesis, upregulates NF-κB signaling, and enhances the expression of its downstream target Bace1, suggesting that altered DNA methylation modulates the expression of genes governing neuronal development, synaptic function, and neuroinflammation (53). Furthermore, DNA methylation signatures in cord blood at birth can predict the genetic susceptibility to neurodevelopmental disorders (including ASD, ADHD, and schizophrenia), demonstrating that prenatal environmental factors interact with genetic backgrounds to establish early-life epigenetic landscapes (54).

Histone modifications also participate critically in developmental epigenetic regulation. Epigenetic modifications, including DNA methylation and histone remodeling, dynamically shape and stabilize cellular epigenomic profiles to control spatiotemporal gene expression (55). During neurogenesis, acetylation and methylation of core histones modulate chromatin accessibility, thereby determining neural stem cell fate and neuronal functional maturation. Accumulating evidence has identified aberrant histone modification patterns in the prefrontal cortex, amygdala, and HPA-axis-associated neural circuits in multiple anxiety-like phenotypes, alongside disrupted GABAergic and glutamatergic signaling transduction (56). Prenatal e-cigarette aerosol exposure induces sex-biased locus-specific DNA methylation alterations (covering CpG and CH sites) and transcriptomic remodeling in the neonatal brain, disrupting neuronal projection, axonogenesis, and signaling pathways linked to neurodegeneration and chronic depression (57). Collectively, these prenatal epigenetic alterations constitute the fundamental molecular basis linking intrauterine environmental stressors to long-term neurodevelopmental deficits and disease susceptibility.

### 4.2 Oxidative stress and mitochondrial dysfunction

Prenatal exposure-induced oxidative stress and mitochondrial dysfunction represent another core mechanism underlying impaired fetal neurodevelopment and elevated late-life neurodegenerative risk. Environmental toxicants disrupt fetal cerebral redox homeostasis through direct and indirect pathways. Gestational PM2.5 exposure reduces maternal circulating FT3 and FT4 levels, and thyroid dysfunction is tightly coupled with exacerbated oxidative stress (24). Similarly, prenatal exposure to endocrine disruptors including bisphenol A and PCBs impairs neurodevelopment via metabolic perturbation and excessive reactive oxygen species (ROS) generation (58). Environmental toxicants activate maternal and placental NADPH oxidase, triggering robust ROS overproduction, which subsequently induces lipid peroxidation, protein denaturation, and DNA damage in fetal neurons.

As the primary organelle for energy metabolism and ROS generation, mitochondria are particularly vulnerable to oxidative injury. Disrupted mitochondrial dynamics and impaired biogenesis are hallmark early adverse outcomes. Mitochondrial dysfunction and redox imbalance are shared pathological features of multiple chronic human diseases (59). Placental tissues from selective fetal growth restriction cases exhibit severe mitochondrial damage, excessive ROS accumulation, reduced ATP production, compensatory mitochondrial copy number elevation, and decreased COX I expression, indicating the eventual failure of mitochondrial adaptive compensation (60). Critically, such prenatal mitochondrial deficits persist into adulthood and predispose individuals to age-related mitochondrial exhaustion and neurodegeneration. In AD pathogenesis, mitochondrial dysfunction, impaired mitochondrial dynamics, and defective mitophagy are well-recognized core pathological drivers (61). *In vitro* experiments have demonstrated that prenatal exposure to imidacloprid, a typical neonicotinoid pesticide, induces mitochondrial dysfunction characterized by reduced ATP synthesis, collapsed mitochondrial membrane potential, and excessive ROS and hydrogen peroxide release, ultimately leading to DNA damage and neuronal apoptosis (62). Oxidative stress and mitochondrial dysfunction form a self-amplifying positive feedback loop: excess ROS further disrupt mitochondrial electron transport chain function, suppress ATP generation, and imbalance mitochondrial fusion–fission dynamics, ultimately compromising neuronal survival, synaptic plasticity, and global brain function across the lifespan.

### 4.3 Persistent activation of neuroimmunity and neuroinflammation

Prenatal environmental exposures reshape fetal neuroimmune programming and induce persistent low-grade neuroinflammation, thereby disrupting neurodevelopment and increasing lifelong neurodegenerative susceptibility. Microglia, the resident innate immune cells of the central nervous system, serve as the central effector of this developmental immune programming. Prenatal interferon-α exposure induces robust autism-like neurobehavioral deficits in offspring, characterized by reduced social interaction, hippocampal and cerebellar neuronal loss, reactive astrogliosis (elevated GFAP immunoreactivity), reduced brain-derived neurotrophic factor expression, and increased TNF-α levels (63). These findings confirm that prenatal immune challenges induce persistent functional priming of fetal microglia, rendering these cells hyper-responsive to secondary inflammatory stimuli in adulthood and exacerbating progressive neurodegeneration.

Neuroimmune dysregulation and chronic neuroinflammation are central pathological drivers of multiple neurological disorders (64). Compromised blood–brain barrier (BBB) integrity further sustains neuroinflammatory activation. Early-life inflammatory perturbation disrupts BBB developmental maturation and functional integrity. BBB dysfunction is a critical component of AD pathogenesis and synergistically interacts with APOE4 polymorphism and type 2 diabetes to accelerate neurodegeneration (65). Prenatal environmental toxicant exposure impairs fetal BBB formation; for example, gestational benzene exposure induces persistent microgliosis in the offspring hypothalamus, a hallmark of sustained microglial activation (66). Impaired BBB permeability allows peripheral pro-inflammatory cytokines to infiltrate the central nervous system in adulthood, establishing a chronic low-grade neuroinflammatory microenvironment, which is a shared pathological characteristic of nearly all neurodegenerative diseases.

In spinal cord injury and multiple sclerosis, uncontrolled neuroinflammation drives secondary neuronal degeneration, demyelination, and axonal damage via crosstalk between innate and adaptive immune systems (67, 68). Similarly, prenatal maternal infection, psychological stress, or chemical exposure activates maternal immune responses and elevates circulating pro-inflammatory cytokines (e.g., IL-1β, IL-6, and TNF-α). These inflammatory mediators either cross the placental barrier or disrupt placental endocrine and immune function, thereby remodeling fetal cerebral immune homeostasis, priming microglial reactivity, and impairing developmental BBB maturation (69). This developmental immune priming renders the mature brain extremely vulnerable to secondary pathogenic insults (e.g., infection, trauma, and aging), triggering exaggerated neuroinflammatory cascades and accelerating neurodegenerative progression in later life.

### 4.4 Crosstalk and integration of core pathological mechanisms

The four aforementioned core mechanisms do not function independently or linearly; instead, they form a highly synergistic and self-amplifying pathological network via interactive regulation, cascade amplification, and multiple positive feedback loops. Epigenetic reprogramming acts as the upstream initiator and biological memory module, suppressing the transcription of antioxidant, anti-inflammatory, and neuroprotective genes and rendering developing neurons intrinsically vulnerable to external insults. Excessive ROS derived from environmental exposure directly disrupts mitochondrial structure and electron transport chain function, triggering severe mitochondrial dysfunction; damaged mitochondria further release massive ROS, establishing a vicious cycle of oxidative stress and mitochondrial failure. Sustained redox imbalance and mitochondrial damage subsequently trigger microglial activation and chronic neuroinflammation. In turn, inflammatory signaling reinforces epigenetic silencing of protective genes and exacerbates oxidative stress, ultimately consolidating transient intrauterine environmental perturbation into stable, long-term abnormal neurodevelopmental phenotypes (Table 2).

**Table 2:** Core mechanisms and key targets underlying neurodevelopmental impairments induced by prenatal environmental exposure.

Core mechanism	Major manifestations	Key regulatory targets/molecules	Effects on neurodevelopment	Relevance to neurodegenerative diseases
Epigenetic programming	Aberrant DNA methylation, altered histone modification, and dysregulated miRNA-mediated regulation	*Bace1*, NF-κB, miR-778	Disordered neural stem cell differentiation and impaired synaptic function and maturation	Modulates pathological protein expression and initiates persistent pro-inflammatory pathway activation, predisposing to late-life neurodegeneration
Oxidative stress and mitochondrial dysfunction	Excessive reactive oxygen species production, deficient ATP synthesis, and structural and functional mitochondrial damage	Glutathione, COX I, NADPH oxidase	Neuronal DNA oxidative damage and accelerated neuronal apoptosis	Facilitates premature brain aging and promotes abnormal misfolding and aggregation of neurodegeneration-associated pathological proteins
Sustained neuroinflammatory activation	Microglial priming and hyperactivation, elevated pro-inflammatory cytokines, and compromised blood–brain barrier integrity	TNF-α, IL-6, BDNF, GFAP	Progressive neuronal loss and disrupted myelination and neural circuit remodeling	Aggravates dopaminergic neuronal injury and exacerbates chronic neuroinflammatory degeneration, augmenting the susceptibility to Parkinson's disease and other neurodegenerative disorders

**Figure 1:**
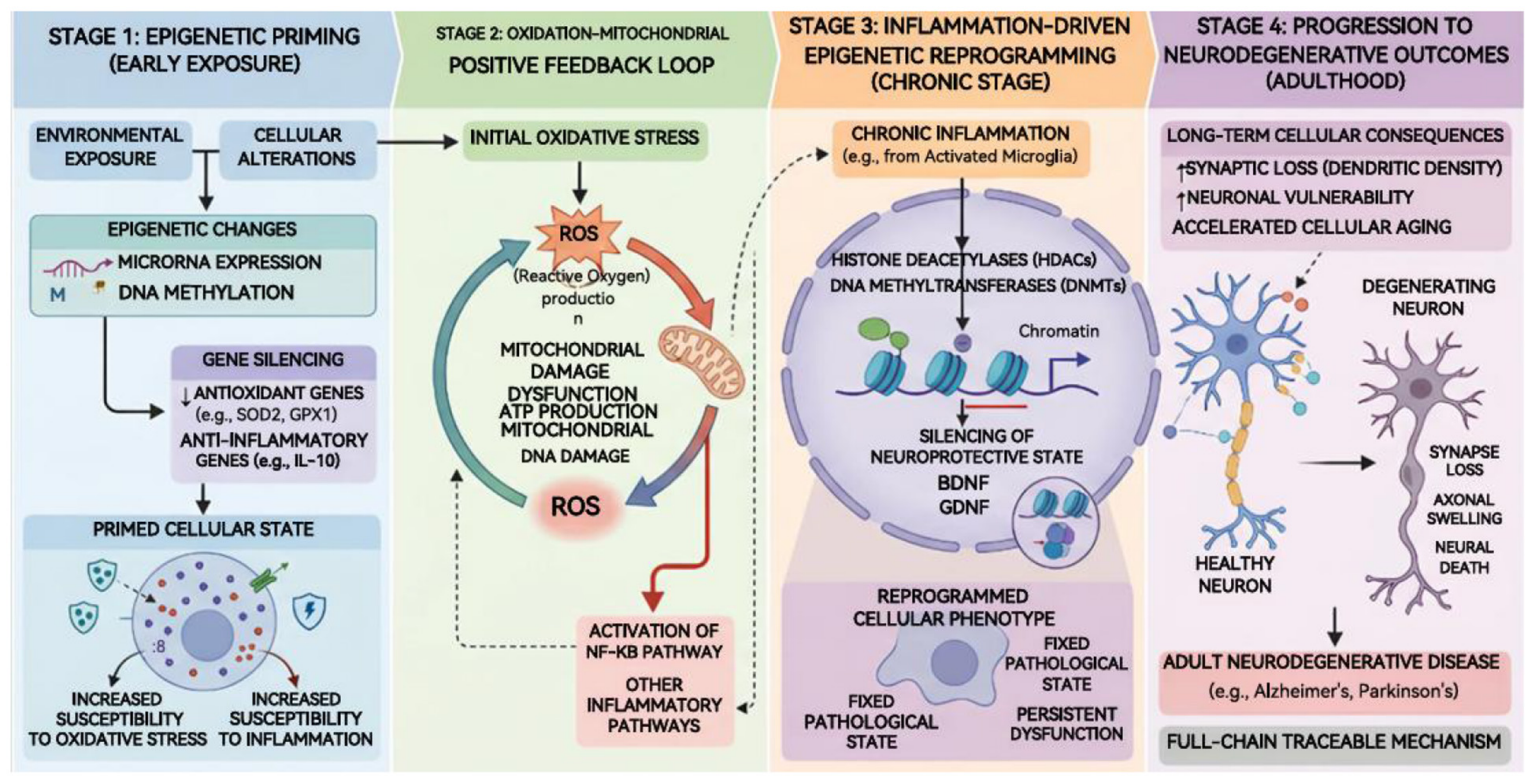
Crosstalk and interaction of core mechanisms.

The synergistic interactions among these mechanisms persistently impair synaptic plasticity, accelerate brain senescence, and reduce neuronal stress resistance and survival capacity. This integrated multi-pathway network fundamentally explains how prenatal environmental exposure rewires neurodevelopmental trajectories and programs lifelong susceptibility to adult-onset neurodegenerative diseases (Figure 1).

## 5 Interactive effects and individual differences: gene–environment interaction and sexual dimorphism

### 5.1 Modulatory role of genetic susceptibility

The neurodevelopmental impairments and elevated neurodegenerative susceptibility induced by prenatal environmental exposure vary substantially among individuals, which is largely modulated by intrinsic genetic backgrounds. Gene–environment (G × E) interaction is a core determinant explaining individual heterogeneity in complex neurological diseases (70). Offspring carrying specific genetic variants associated with neurodegenerative disorders exhibit heightened vulnerability to prenatal environmental insults. The apolipoprotein E (APOE) ε4 allele is the most robust genetic risk factor for Alzheimer's disease (AD). Accumulating evidence has demonstrated that the associations between plasma homocysteine levels and neurodegenerative biomarkers (e.g., neurofilament light chain and phosphorylated tau) are more pronounced in APOE ε4 non-carriers, indicating that genetic background dictates the magnitude of environmental and metabolic contributions to neuropathological progression (71). For Parkinson's disease (PD), large-scale studies integrating polygenic risk scores and common GBA variants have explored the interactions between genetic susceptibility and multiple environmental and lifestyle factors (e.g., type 2 diabetes, physical activity, body mass index, and tobacco use), confirming that genotypes significantly modify the environmental risks of PD (72). These findings provide preliminary evidence illustrating how prenatal environmental exposure interacts with inherent genetic vulnerability to accelerate or reshape the pathological trajectory of neurodegenerative diseases.

Large-scale genome-wide association studies (GWAS) have been increasingly applied to identify genome-wide G × E interactions and novel susceptible genetic loci related to prenatal exposure outcomes (73). Traditional GWAS primarily prioritize common genetic variants associated with disease phenotypes, whereas G × E analyses further clarify context-dependent genetic effects under diverse environmental conditions (74). In complex disorders including cardiovascular diseases, type 2 diabetes, and major depressive disorder, GWAS have identified numerous risk loci, and subsequent G × E studies have elucidated how these genetic vulnerabilities interact with exogenous stressors (e.g., smoking, dietary patterns, and early-life adversity) to collectively drive disease pathogenesis (75–77). In the field of neurodegeneration, preliminary investigations into progressive supranuclear palsy have revealed potential interactions betweenMAPT and EIF2AK3 loci and occupational and agricultural environmental risks, although these associations require validation in larger cohorts (78). These studies advance our understanding of the genetic basis underlying individual differences in neurodevelopmental outcomes following prenatal environmental exposure. With the rapid advancement of large-scale biobanks and sophisticated statistical approaches (e.g., penalized variable selection and deep learning models), the analytical capacity for G × E interaction detection has been substantially improved, facilitating precise disease risk prediction and supporting the development of individualized preventive and therapeutic strategies (74, 79).

### 5.2 Sex-specific effects

Prenatal environmental exposure exerts pronounced sexually dimorphic effects on offspring neurodevelopment, which stem from inherent sex-based disparities in brain maturation and the regulatory actions of sex hormones. Brain sexual differentiation is a sophisticated developmental process governed by sex chromosome complement (XX vs. XY) and the organizational effects of steroid hormones (e.g., androgens and estrogens), which collectively shape the structural and functional heterogeneity of male and female brains (80, 81). Transcriptomic differences between male and female brains emerge as early as embryonic development, prior to the activation of gonadal hormones, suggesting that sex chromosome-linked genes (e.g., SRY and EIF2S3Y on the Y chromosome) directly participate in early cerebral sexual differentiation (82). During adolescence and adulthood, the activational effects of sex hormones further modulate neuroimmunity, synaptic plasticity, and other core neural processes, leading to intrinsic sexual disparities in neuroinflammatory responses and dopaminergic circuit programming (83). As resident innate immune cells in the central nervous system, microglia exhibit sex-specific gene expression profiles and functional phenotypes, which are dynamically regulated by sex hormones and critically determine sexual dimorphism in social behaviors, reproductive functions, and neurodegenerative susceptibility (83). The male predominance of autism spectrum disorder is attributable to the combined effects of prenatal androgen exposure, sex chromosome genetics, and crosstalk between sex hormones and immune systems, which drive sex-biased neurodevelopmental trajectories (84).

Sex disparities in prenatal exposure outcomes are further attributed to differential epigenetic regulation of X-chromosomal genes. In female mammals, one of the two X chromosomes undergoes X chromosome inactivation (XCI) during early embryonic development, a long non-coding RNA Xist-mediated epigenetic silencing mechanism that balances X-linked gene dosage between sexes (85, 86). Nevertheless, over 15% of X-linked genes escape complete XCI and maintain expression from the inactive X chromosome, resulting in inherent sex differences in X-linked gene dosage (84). Such escape phenomena are gene-specific and individually variable, tightly modulated by epigenetic marks including DNA methylation and histone modifications (84). Genome-wide differential DNA methylation profiles at promoter regions of inactive X chromosomes have been identified in females relative to males, which are strongly correlated with XCI escape status (87). Accordingly, identical prenatal environmental insults may induce distinct long-term neurodevelopmental consequences in male and female offspring by interfering with the epigenetic regulation of X-linked genes, most of which are critical for neurodevelopment, immune homeostasis, and metabolic function. In females, environmental stressors may disrupt the establishment or maintenance of XCI and remodel the expression patterns of escape genes, thereby altering neurodevelopmental trajectories and neurodegenerative susceptibility in a sex-specific manner (88, 89). A comprehensive understanding of the complex regulatory network orchestrated by sex chromosomes and sex hormones is essential to elucidate the mechanistic basis of sexual dimorphism in the health impacts of prenatal environmental exposure.

## 6 Research methodologies and model systems

### 6.1 Epidemiological studies and birth cohorts

Prospective birth cohort studies represent the most robust epidemiological approach to delineating the causal associations between prenatal environmental exposure and offspring neurodevelopmental outcomes as well as long-term neurodegenerative susceptibility. By longitudinally collecting detailed exposure data, neuroimaging parameters, behavioral assessment results, and biological samples across critical gestational and postnatal developmental stages, prospective cohorts establish temporally ordered exposure–outcome relationships and provide rigorous evidence for causal inference. The Dutch Hunger Winter Cohort, for instance, has systematically revealed the long-term impacts of acute gestational malnutrition on cardiovascular, metabolic, and cerebral health across the lifespan, with outcomes highly dependent on the specific gestational window of exposure (90). Similarly, the Shanghai Pregnancy and Sleep Cohort in China prospectively records late-gestation sleep parameters and conducts up to 6 years of pediatric follow-ups, aiming to clarify the influences of prenatal sleep disturbances on child growth and neuropsychiatric development (91). Large-scale international cohorts, such as the Boston Birth Cohort, enroll substantial populations of preterm infants and their mothers to construct integrated gene–environment databases, uncovering the early-life origins of preterm birth and chronic pediatric diseases (92). The prospective design minimizes recall bias and captures dynamic alterations in environmental exposure and developmental phenotypes, providing irreplaceable evidence for elucidating the developmental programming of long-term neurological health by prenatal environmental factors.

Retrospective studies and Mendelian randomization (MR) analyses serve as critical complementary tools for causal inference in prenatal exposure-related neurodevelopmental research. Retrospective observational analyses based on archived medical records and historical datasets exhibit unique advantages in investigating rare neurological outcomes and leveraging existing large-scale population databases, despite inherent limitations including recall bias and residual confounding (93). To overcome the confounding effects pervasive in observational studies, MR has emerged as a powerful analytical strategy. This method utilizes genetic variants strongly associated with environmental exposures or maternal biomarkers as instrumental variables. Given that genotypes are randomly allocated at conception and unaffected by postnatal environmental confounding factors, MR enables more reliable causal evaluation of exposure–outcome associations (94). For example, genetic variants linked to maternal inflammatory biomarkers and nutritional status can be applied to infer the causal effects of gestational inflammation and malnutrition on offspring neurodevelopment (95). Multivariable MR further accommodates multiple correlated exposures and mediating pathways, enabling refined dissection of the causal mechanisms underlying prenatal environmental programming of neurological phenotypes (96). Despite inherent methodological assumptions (e.g., independence, relevance, and exclusion restriction of instrumental variables) and potential pleiotropic effects, MR combined with prospective cohort evidence significantly strengthens the robustness of causal inference, providing credible theoretical support for public health intervention formulation (97).

### 6.2 Experimental animal models and cellular systems

Transgenic animal models provide a controllable experimental platform to directly verify whether specific prenatal environmental exposures accelerate or exacerbate neurodegenerative pathological phenotypes in offspring, enabling precise exploration of G × E interactions. Prenatal environmental interventions in female transgenic animals carrying human disease-associated genes effectively simulate the synergistic effects of genetic predisposition and environmental stressors. For instance, prenatal exposure to ambient fine particulate matter or psychological stress in AD-model APP/PS1 mice can directly induce premature or aggravated Aβ deposition, tau hyperphosphorylation, and cognitive deficits in offspring (98). Similarly, prenatal toxicological intervention in PD or amyotrophic lateral sclerosis transgenic models helps clarify how environmental insults interact with genetic susceptibility to modulate the survival and functional integrity of dopaminergic and motor neurons (99). These *in vivo* models allow comprehensive mechanistic dissection of exposure-induced pathological alterations at molecular, cellular, and tissue levels, including neuroinflammation, oxidative stress, mitochondrial dysfunction, and synaptic remodeling (100). Furthermore, conditional knockout and overexpression transgenic models enable targeted exploration of the specific roles of distinct cell types (e.g., microglia and astrocytes) and signaling pathways in mediating prenatal neurotoxic effects (101). Although animal models cannot fully recapitulate the complexity of human neurological diseases due to interspecies differences, they are indispensable for establishing causal chains, identifying early predictive biomarkers, and validating potential therapeutic and preventive targets.

Human brain organoids and *in vitro* culture systems have revolutionized the mechanistic investigation of prenatal environmental neurotoxicity under highly controllable experimental conditions. Human pluripotent stem cell (hPSC)-derived brain organoids recapitulate the three-dimensional structural architecture and cellular heterogeneity of the developing human cerebral cortex, enabling precise evaluation of how environmental toxicants (e.g., heavy metals and persistent organic pollutants) and nutritional factors modulate neural progenitor proliferation, differentiation, migration, and neural network formation (102). Exposure of brain organoids to gradient concentrations of environmental chemicals facilitates the assessment of toxic effects on neuronal survival, synaptogenesis, and glial function, with transcriptomic alterations further characterized via single-cell RNA sequencing and other high-throughput technologies (103). Patient-derived hPSCs can be differentiated into disease-specific brain organoids to recapitulate the pathological features of neurodegenerative disorders and verify whether environmental exposure exacerbates disease-related phenotypes (104). In addition, co-culture systems of primary neurons (e.g., cortical neurons) and glial cells (e.g., astrocytes and microglia) allow refined exploration of cell–cell crosstalk in mediating environmental neurotoxicity (105). These human-derived *in vitro* systems eliminate the uncertainty of interspecies extrapolation and support high-throughput compound screening and mechanistic validation (106). Nevertheless, current brain organoid models possess inherent limitations in simulating intact blood–brain barrier structure, sophisticated neural circuits, and long-term aging processes. Future advancements integrating bioengineering technologies (e.g., organ-on-a-chip systems) will optimize the simulation of *in vivo* physiological microenvironments and dynamic gestational exposure processes, enabling more accurate risk assessment of prenatal environmental exposure-induced long-term neurological deficits in human offspring (107) (Table 3).

**Table 3:** Summary of animal experimental studies.

No.	First author	Year	Model/intervention	Main findings
(1)	Ge C	2021	Placental P-glycoprotein (P-gp) inhibition	Inhibition of placental P-gp opens the placental glucocorticoid barrier, leading to reduced fetal body weight.
(7)	Baines KJ	2020	Maternal immune activation in rats	Maternal immune activation alters fetal brain development and promotes the proliferation of neural progenitor cells.
(10)	Dali O	2024	Nicotine exposure in rat testis	Nicotine exposure induces epigenetic alterations in genes associated with peripheral nervous system signaling.
(41)	Hao F	2024	Gestational pyrethroid exposure in mice	Gestational pyrethroid exposure impairs neurodevelopment in male offspring.
(42)	Tian L	2024	Gestational nanoplastics exposure in rats	Maternal nanoplastics exposure disrupts neurodevelopment in rat offspring.
(44)	Fagundes BHF	2022	Methylmercury exposure in rats	Methylmercury exposure triggers oxidative stress and results in motor and cognitive impairments.
(49)	Loan A	2023	Methylmercury exposure in mice	Methylmercury exposure causes premature neuronal differentiation and autism-like behavioral phenotypes.
(51)	Rather HA	2022	Full-gestation cocaine exposure in rhesus monkeys	Proteomic profiling was performed on cerebrospinal fluid extracellular vesicles from exposed rhesus monkeys.
(63)	Otkiran G	2026	Interferon-α exposure in male offspring	Interferon-α exposure induces autism-like neurobehavioral deficits and neurochemical abnormalities.
(66)	Koshko L	2023	Amphetamine exposure in mice	Prenatal amphetamine exposure leads to hypothalamic developmental defects and increases susceptibility to metabolic disorders.

## 7 Prevention, intervention and translational perspectives

### 7.1 Risk-based early screening and surveillance

The identification of robust biomarkers that reflect prenatal environmental toxic effects and predict offspring neurodevelopmental risks is pivotal for early warning and precise intervention. Current research primarily focuses on screening specific molecular biomarkers from placental tissue, umbilical cord blood, and maternal peripheral blood. Accumulating evidence indicates that prenatal phthalate (PAE) exposure disrupts maternal thyroid hormone homeostasis. Multiple PAE metabolites, including monomethyl phthalate (MMP) and mono(2-ethylhexyl) phthalate (MEHP), are significantly correlated with altered levels of total thyroxine (TT4), free triiodothyronine (FT3), and free thyroxine (FT4), suggesting that maternal thyroid hormone profiles serve as promising biomarkers for evaluating the endocrine-disrupting effects of environmental toxicants (27).

Furthermore, the application of non-targeted analysis (NTA) has substantially expanded the landscape of identifiable prenatal chemical exposures beyond conventional targeted detection approaches. A recent study utilized NTA to qualitatively and quantitatively characterize exogenous and endogenous chemicals in maternal and cord blood, detecting pervasive pollutants including perfluorooctane sulfonate (PFOS) and perfluorohexane sulfonate (PFHxS). Linear PFOS, PFHxS, octadecanedioic acid, and deoxycholic acid were identified in over 97% of maternal samples, and their elevated concentrations were closely associated with increased risks of gestational diabetes mellitus (GDM) and hypertensive disorders of pregnancy, highlighting their potential as candidate biomarkers for adverse gestational outcomes and subsequent long-term neurological deficits (108). Another exposome-based study integrated untargeted high-resolution mass spectrometry data of prenatal biological specimens and identified suspect environmental chemicals linked to long-term disease risks, including N-substituted piperidine insecticides and 2,4-dinitrophenol associated with subsequent breast cancer susceptibility (109). This framework provides a novel strategy for exploring the mechanistic links between prenatal environmental insults and distant offspring disorders, including neuroendocrine-related malignancies. Collectively, the combination of conventional targeted detection and emerging non-targeted omics technologies enables systematic screening and validation of circulating microRNAs, oxidative damage products, inflammatory mediators, and toxic metabolites, laying a critical foundation for constructing predictive models of prenatal environmental neurotoxicity risks.

Advanced neuroimaging techniques offer another promising strategy for the early detection of subtle structural and functional brain abnormalities in vulnerable children. Although existing neuroimaging biomarkers are predominantly established for adult neurodegenerative diseases, their technical principles and analytical paradigms are adaptable to pediatric early warning surveillance. For instance, plasma phosphorylated tau217 (p-tau217) exhibits strong correlations with amyloid positron emission tomography (PET) signals and closely tracks cognitive deterioration and brain structural atrophy in Alzheimer's disease, validating its high diagnostic and predictive value (110). These findings suggest that integrating validated prenatal exposure-related humoral biomarkers with neuroimaging phenotypes can significantly improve the accuracy of early risk prediction for late-life neurodegeneration.

Pediatric neuroimaging technologies have undergone substantial advancements, enabling precise evaluation of typical brain developmental trajectories and early identification of neurodevelopmental disorders (111). Future cohort studies focusing on children with documented high-risk prenatal exposures (e.g., ambient air pollution and specific chemical toxicants) are warranted to explore the utility of diffusion tensor imaging (DTI) for assessing white matter tract integrity and resting-state functional magnetic resonance imaging (rs-fMRI) for evaluating cerebral network connectivity. These neuroimaging modalities can detect subtle deviations in neurodevelopment prior to the onset of clinical symptoms. Innovative analytical frameworks combining environmental mixture exposure data, biological knowledge graph-based networks, and machine learning algorithms have been successfully applied to predict early pregnancy loss (112). Analogous multi-omics and neuroimaging integrative strategies hold great potential for screening high-risk individuals with prenatal exposure-induced neurodevelopmental dysregulation, thereby facilitating ultra-early warning and targeted precision intervention.

### 7.2 Potential intervention strategies

Optimization of prenatal nutritional status and lifestyle represents a feasible and effective intervention to mitigate the adverse neurodevelopmental impacts of environmental toxicants. Adequate nutritional supply is essential for antagonizing oxidative and inflammatory damage induced by exogenous pollutants. Notably, healthy dietary patterns, including the Alternative Mediterranean Diet and Alternative Healthy Eating Index, confer cardiovascular and metabolic benefits but may simultaneously increase gestational exposure to persistent organic pollutants such as polychlorinated biphenyls (PCBs) and per- and polyfluoroalkyl substances (PFAS) via dietary intake (29). This paradox underscores the urgent need for coordinated environmental governance to reduce food chain contamination while advocating healthy prenatal diets.

Targeted nutritional supplementation exerts protective effects against prenatal environmental stressors. Gestational iodine sufficiency is indispensable for fetal neurodevelopment, and systematic iodine monitoring and standardized supplementation for pregnant women are essential clinical preventive measures (113). Personalized nutritional counseling has been validated to optimize gestational nutritional status. In a cohort of pregnant women with prior bariatric surgery, individualized nutritional intervention significantly improved dietary and supplemental intake of calcium and iron, contributing to normalized neonatal birth weight (114). Beyond nutritional regulation, active avoidance of harmful environmental exposures is a core lifestyle intervention. Prenatal environmental tobacco smoke (ETS) exposure significantly increases the incidence of gestational complications (53.7% vs. 42.3%) and adverse birth outcomes such as low Apgar scores (115). Additionally, maternal exposure to benzophenones (BPs) and other endocrine-disrupting chemicals is closely correlated with personal care product usage, dietary habits, and physical activity levels (28). Accordingly, targeted health education should guide pregnant women to reduce the use of chemical-containing personal care products, optimize food storage practices, and maintain appropriate physical activity. A full-cycle guidance system covering pre-pregnancy preparation, gestational health management, and postpartum recovery is critical for optimizing maternal and child health and buffering environmental exposure-related risks (116).

Targeting stable epigenetic modifications induced by early-life environmental programming holds great promise for developing novel epigenetic reprogramming interventions to reverse adverse developmental alterations within critical therapeutic windows. Epigenetic signatures, including DNA methylation and histone modification, are environmentally sensitive regulators of gene transcription. Prenatal exposure to environmental endocrine disruptors (EEDs) remodels fetal epigenetic landscapes, thereby programming long-term disease susceptibility and even elevating the risk of pediatric germ cell tumors (117). Accumulating translational progress in epigenetic therapy for malignancies provides robust theoretical support for neurodevelopmental intervention. DNA methyltransferase inhibitors (e.g., azacitidine and decitabine) and histone deacetylase inhibitors (HDACis) have been widely applied to reverse aberrant epigenetic silencing and restore normal cellular function in tumor treatment (118).

Drawing on these advances, future research can explore low-dose, high-safety epigenetic modulators to precisely reverse exposure-induced epigenetic aberrations at neurodevelopment- and neurodegeneration-associated loci during critical developmental windows (e.g., early childhood and pre-adolescence). Preclinical studies have validated that HDACis upregulate major histocompatibility complex class I (MHC-I) expression in neuroblastoma cells and enhance cellular immunogenicity, confirming the regulatory potential of epigenetic agents in modulating neuronal function and homeostasis (119). Nevertheless, translational application of epigenetic reprogramming for neurodevelopmental protection faces substantial challenges, including the requirement for ultra-high targeting specificity to avoid global epigenomic dysregulation and the need to define safe and effective intervention time windows. Current priority research should focus on elucidating the precise mechanisms by which specific environmental toxicants induce locus-specific epigenetic alterations in neurodevelopmental genes and identifying nutritionally or pharmacologically modifiable key targets, laying a foundation for future precision epigenetic intervention strategies.

A full-life-cycle prevention and management system covering pre-pregnancy counseling, gestational healthcare, pediatric neurodevelopmental surveillance, and adult neurological health monitoring requires multidisciplinary collaboration integrating obstetrics, pediatrics, neurology, and environmental medicine. The multidisciplinary team (MDT) model has been proven to optimize clinical outcomes in complex chronic disease management. For example, MDT intervention combining hepatologists, psychiatrists, addiction counselors, and social workers enables comprehensive dual-pathway management for alcoholic liver disease and alcohol use disorders (120). Similarly, prenatal environmental exposure-induced neurodevelopmental programming involves reproductive, developmental, neurological, and environmental disciplines, necessitating standardized MDT management.

In the pre-pregnancy stage, environmental physicians and obstetricians collaboratively evaluate familial environmental exposure risks and provide individualized guidance for occupational protection and residential environmental improvement. Accumulating evidence indicates that green space exposure mitigates the adverse impacts of urban environmental stressors such as air pollution and noise during gestation, providing novel environmental intervention insights for prenatal healthcare (23). During pregnancy, multidisciplinary teams including professional nutritionists deliver personalized nutritional and lifestyle guidance to buffer toxic exposure risks (113). Postnatally, routine pediatric follow-ups should incorporate standardized neurodevelopmental assessment and early neuroimaging screening for high-risk infants with confirmed prenatal toxic exposure history, enabling timely referral and early behavioral intervention once developmental deviations are identified. Furthermore, long-term prospective cohorts jointly supervised by neurologists, epidemiologists, and environmental experts are essential for tracking neurodegenerative susceptibility in high-risk populations and validating the long-term efficacy of early interventions. This integrated prevention–treatment–management model breaks disciplinary barriers, establishes standardized collaborative workflows and information-sharing platforms, and realizes dynamic risk assessment and proactive whole-life neurohealth management.

## 8 Conclusion

Prenatal environmental exposure represents a critical developmental programming window that profoundly shapes offspring neurodevelopmental trajectories and lifelong susceptibility to neurodegenerative diseases. Compelling evidence confirms that diverse gestational environmental stressors interact synergistically rather than functioning independently. They remodel fetal cerebral developmental landscapes via intertwined pathological cascades, including epigenetic reprogramming, persistent oxidative stress, mitochondrial dysfunction, and chronic neuroinflammation, ultimately determining long-term neurological health and disease vulnerability. These findings advance the developmental origins of health and disease (DOHaD) theory from conceptual speculation to mechanistic specificity.

Notably, substantial individual heterogeneity exists in the neurodevelopmental outcomes of prenatal environmental exposure. Genetic background and sex serve as critical effect modifiers, driving divergent phenotypic responses to identical toxic insults. This evidence strongly suggests that future public health risk assessment and intervention strategies should abandon the one-size-fits-all paradigm and shift toward precise, individualized prevention and management. Negative findings from eligible studies do not negate the programming effects of prenatal environmental stressors but reflect contextual differences in population characteristics, exposure dosage, and evaluation time points. Integration of seemingly contradictory empirical data facilitates the construction of a comprehensive and dynamic exposure–response framework and clarifies the boundary conditions of environmental developmental toxicity.

Future breakthroughs in this field rely on innovative research paradigms and refined mechanistic exploration. First, multi-omics integrative analysis combined with large-scale longitudinal birth cohorts is urgently needed to dissect the causal chains linking specific prenatal exposures, precise molecular mechanisms, and definite neurodevelopmental phenotypes, thereby identifying reliable biomarkers for early warning and risk stratification. Second, mechanistic research should transcend single-pathway description and prioritize the crosstalk between epigenetic, inflammatory, and metabolic regulatory networks to uncover core actionable intervention targets. In addition, although prospective human cohorts have validated the associations between prenatal environmental exposure and pediatric neurodevelopmental deficits, direct longitudinal human evidence confirming the causal relationship between gestational insults and adult-onset Alzheimer's and Parkinson's diseases remains insufficient, with current inferences largely speculative. Future studies should emphasize multi-mechanistic interaction, long-term longitudinal follow-up, and precise translational intervention. Rigorous translational principles are required to avoid overextrapolation of preclinical animal data and supraphysiological experimental results to real-world human exposure scenarios, providing solid and credible theoretical foundations for public health policy formulation.

Future public health strategies require dual-track optimization of primary prevention and early intervention. Fundamentally, strengthened environmental governance is essential to reduce gestational harmful exposure sources at the root. Meanwhile, evidence-based early interventions, including standardized nutritional supplementation (e.g., folic acid and specific polyunsaturated fatty acids) and scientific lifestyle guidance, should be widely promoted. Shifting the preventive frontier of neurodevelopmental disorders and neurodegenerative diseases forward to the gestational period will fundamentally reshape disease epidemiological trajectories and build resilient neurodevelopmental trajectories for fetal and pediatric brain health across the lifespan.
